# The blood purification therapy-based strategy effectively rescued the severe falciparum malaria patient experiencing cytokine storm-driven MODS

**DOI:** 10.3389/fcimb.2025.1682892

**Published:** 2026-01-02

**Authors:** Ying Sun, Xi Huang, Bao Hong, Jinyuan Song, Ying Lu, Jindi Ma, Xiaojing Wang, Wenqing Hu, Yimin Zhang, Hua Xuan

**Affiliations:** 1 State Key Laboratory for Diagnosis and Treatment of Infectious Diseases, National Clinical Research Center for Infectious Diseases, National Medical Center for Infectious Diseases, The First Affiliated Hospital, Zhejiang University School of Medicine, Hangzhou, China; 2 Department of Electrocardiogram, The First Affiliated Hospital, School of Medicine, Zhejiang University, Hangzhou, China; 3 Department of Infectious Diseases, The Affiliated Haining Hospital, Jiaxing University, Jiaxing, China; 4 Department of Clinical Laboratory, The Affiliated Haining Hospital, Jiaxing University, Jiaxing, China; 5 Emergency Department, The Affiliated Haining Hospital, Jiaxing University, Jiaxing, China; 6 Department of Infectious Diseases, Affiliated Hospital of Jiaxing University, The First Hospital of Jiaxing, Jiaxing, China

**Keywords:** continuous veno-venous hemodiafiltration, cytokine storm, falciparum malaria, multiple organ dysfunction syndrome, therapeutic plasma exchange

## Abstract

**Background:**

The mortality of severe falciparum malaria is high, in which cytokine storm (CS) plays an important role, and the standard effective therapy strategy remains unclear.

**Methods:**

A 63-year-old severe falciparum malaria patient with CS was treated with therapeutic plasma exchange (TPE) combined with continuous venovenous hemodiafiltration (CVVHDF) at the onset of multiple organ dysfunction syndrome (MODS). All clinical data, including symptoms, laboratory results, and treatment methods during the hospitalization period, were collected from electronic medical records, and the dynamic changes of serum cytokines were recorded simultaneously. At the same time, we searched databases such as PubMed, Web of Science, and Scopus and summarized case reports on the use of blood purification as an adjuvant therapy for patients with severe *Plasmodium* infection.

**Results:**

After the first session of TPE+CVVHDF therapy, aberrant elevated cytokines, especially interleukin-6 and interferon-gamma, decreased sharply from 729.65 to 26.42 pg/mL and 170.38 to 42.47 pg/mL, respectively. *Plasmodium* was controlled, and the A Physiology and Chronic Health Evaluation II score, neutrophil-to-lymphocyte ratio, procalcitonin, and C-reactive protein ameliorated as the patient gradually recovered from MODS. Most of the pro-inflammatory or anti-inflammatory cytokines showed positive relationship trends to parasite count in the recovery stage. Among the case reports of severe malaria infection that we summarized, a total of 29 patients received antimalarial regimens with blood purification as an adjuvant therapy, in addition to six small cohort studies. Of these, 19 patients who received TPE or blood purification treatment, including TPE, all exhibited systemic inflammatory symptoms or developed MODS in the early stage of infection, and all these cases eventually had a good prognosis.

**Conclusion:**

The TPE+CVVHDF-based strategy provided a promising CS approach in rescuing severe falciparum malaria patients with MODS.

## 1 Introduction

Malaria is one of the most important epidemic parasitic infectious diseases in the world, causing more than half a million deaths each year (Suh et al., 2004). Falciparum malaria is an important traveling-related severe malaria that causes death. It rapidly evolves into cerebral malaria and serious malaria and then advances into acute renal failure, severe anemia, bleeding, and various other severe complications, which will cause multiple organ dysfunction syndrome (MODS), even leading to multiple organ failure (MOF) (Botella de Maglia et al., 1995).

Cytokine storm (CS) is triggered by the pathogen to a systemic inflammatory reaction, releasing pro-inflammatory factors and anti-inflammatory factors into circulation (Fajgenbaum and June, 2020; Jarczak and Nierhaus, 2022). More and more evidence showed that cytokines play an important role in the immune response to *Plasmodium falciparum* (Berg et al., 2015). The imbalance of cytokines and failure to control the disease contribute to the pathophysiological development of the disease (Rhee et al., 2001). Timely and effective inhibition of CS has an important influence on the prognosis of the disease. However, few methods have been shown to effectively block CS.

In this study, we reported the CS targeting strategy based on a blood purification regimen—therapeutic plasma exchange (TPE) combined with continuous venovenous hemodiafiltration (CVVHDF), applied in addition to standard antimalaria therapy at the onset of MODS, which successfully rescued the severe falciparum malaria patient with CS. A panel of pro-inflammatory and anti-inflammatory cytokines was tested throughout the course of therapy. The correlation between dynamic changes of cytokines, severity parameters, and *Plasmodium* density was analyzed to evaluate the value of CS control in such progressively lethal *P. falciparum* infection.

## 2 Methods

### 2.1 Case study

The case study was a patient with severe falciparum malaria admitted and diagnosed at the People’s Hospital of Haining City, Zhejiang Province, China, on 28 January 2022. Severe malaria is defined by the involvement of vital organs in the infected individual, including shock, pulmonary edema, significant bleeding, seizures, impaired consciousness, and laboratory abnormalities such as kidney impairment, acidosis, anemia, or high parasitemia (Daily et al., 2022). The patient was treated with an artemisinin-based antimalarial regimen. After admission, the patient developed a cytokine storm and progressed to MODS. The patient received TPE on the second and third days and CVVHDF from the second to the fifth day and was eventually discharged after recovery. This study was approved by the Ethical Review Committee of Haining People’s Hospital (No. 24, 2022), and informed consent was obtained from the patient.

### 2.2 Examination of cytokine concentrations

Residual serum from biochemical examination was frozen at −80°C for cytokine examination. The assay was carried out using the ProcartaPlex Human Th1/Th2 Cytokine Panel 11plex kit (Thermo Fisher Scientific, Waltham, Massachusetts, USA EPX110-10810-901), utilizing the Luminex xMAP technology and according to the manufacturer’s instructions. Cytokine levels were detected using the Luminex 200 system (Luminex, Austin, TX, USA) and analyzed using the ProcartaPlex Analyst v1.0 Software (Thermo Fisher Scientific).

### 2.3 Data collection

All the clinical data, including symptoms, laboratory results, and treatment methods, were collected from the electronic medical records. Blood cell counts were tested using the Sysmex XN-10 (Sysmex Corporation, Japan), serum biochemical indices were tested using the Beckman AU680 (Beckman Coulter, USA), coagulation function was tested using the ACL TOP 750 LAS (Werfen, USA), hypersensitive C-reactive protein (CRP) was tested using the PA990pro (Promen, China), and procalcitonin (PCT) was tested using the Caris200 (XMUMIC Medical Instruments, China).

The study searched for relevant research in multiple electronic databases including PubMed, Web of Science, and Scopus, from 1985 to 2025, including case reports on the use of blood purification as an adjuvant treatment for *Plasmodium* infection. We used various combinations of keywords such as “malaria,” “Plasmodium,” “blood purification,” “plasmapheresis,” “plasma exchange,” “hemoperfusion,” “hemofiltration,” “hemoadsorption,” “renal replacement therapy,” “CRRT,” “CVVH,” “CVVHD,” and “CVVHDF” and finally retrieved 127 relevant studies. After excluding literature that seriously deviated from the theme of this study, had substantial missing or duplicate data, and was not published in English, a total of 35 case reports and small cohort studies were selected.

### 2.4 Statistical analysis

The Spearman correlation test was used to analyze the correlation between cytokine concentrations and *Plasmodium* counts. Statistical tests were performed using R, version 4.0.3 (R Foundation for Statistical Computing, Vienna, Austria). A *P*-value <0.05 was considered statistically significant.

## 3 Results

### 3.1 Diagnosis and treatment course

A 63-year-old Chinese male patient was admitted to the department with a chief complaint of fatigue. He had lived in Africa for a couple of years and left Africa 12 days ago. The patient has a medical history of hypertension, diabetes, and cerebral infarction for more than 10 years, continually taking the corresponding treatment.

On admission, the vital signs were as follows: body temperature, 39.3°C; heart rate, 140 beats/min; respiration, 25 times/min; and blood pressure, 159/98 mmHg. Laboratory findings showed the following results: white blood cell (WBC), 5.8 * 10^9^/L; CRP, 103.9 mg/L; platelet (PLT), 184 * 10^9^/L; alanine transaminase (ALT), 131 U/L; and COVID-19 nucleic acid test, negative. Chest and abdominal computer tomography (CT) showed no obvious abnormal findings. Routine antibiotic medication was given with a nasal oxygen supply.

The situation dramatically deteriorated. Twelve hours after admission, the patient became unconscious and developed MODS. The parameters and clinical symptoms are described in **Supplementary Table S1**.

On the second day, TPE (fresh frozen plasma, 3,000 mL) in combination with CVVHDF was performed with immunoglobulin and methylprednisolone (MP) intravenous injection as additional therapy.

**Figure 1:**
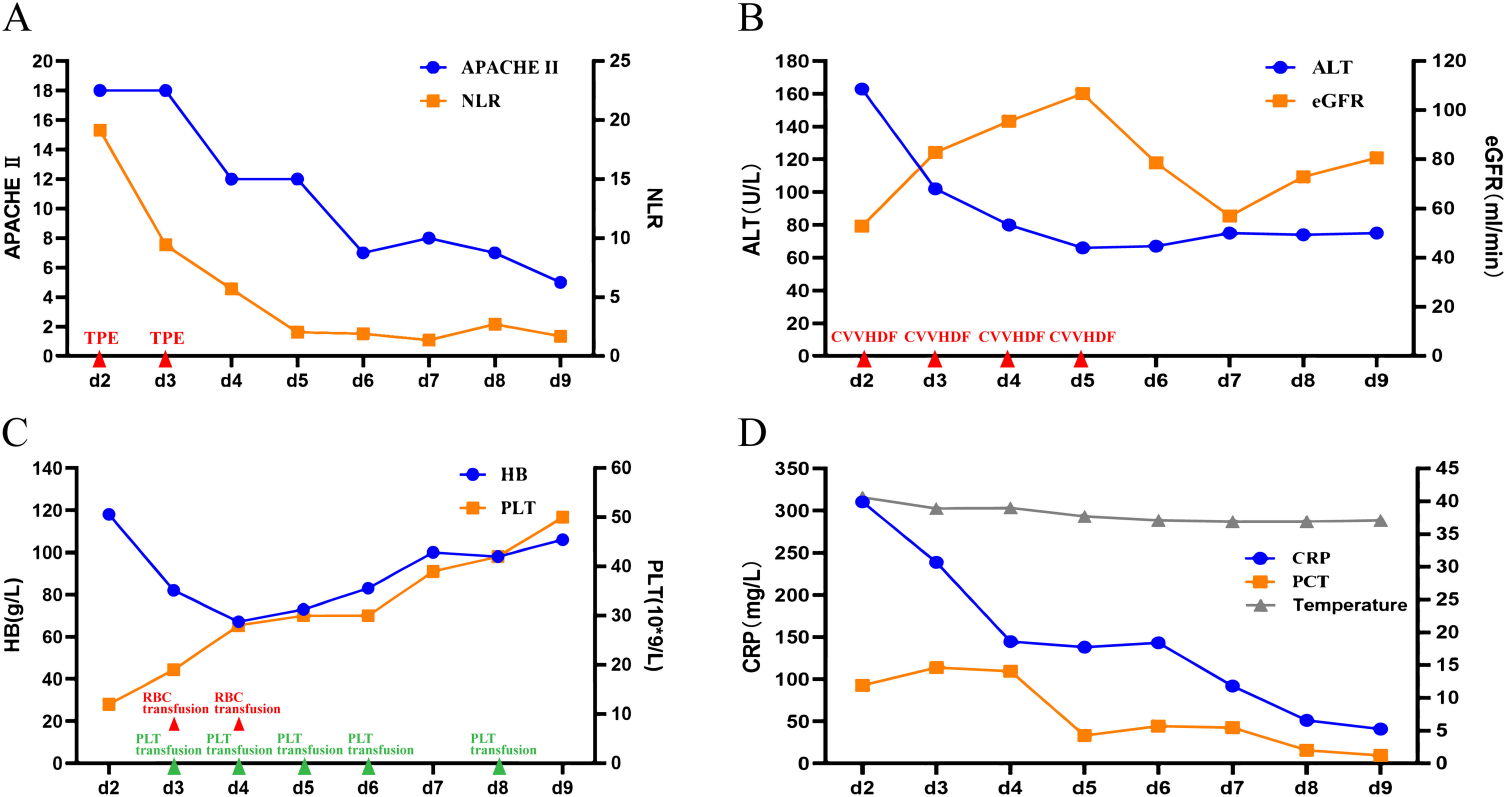
Main treatment course and dynamic changes of key parameters. **(A)** Changes of APACHE II and NLR after admission. **(B)** Changes of ALT and eGFR after admission. **(C)** Changes in HB and PLT after admission. **(D)** Changes of CRP, PCT, and temperature after admission. APACHE II, A Physiology and Chronic Health Evaluation II; NLR, neutrophil-to-lymphocyte ratio; ALT, alanine transaminase; eGFR, estimated glomerular filtration rate; CRP, C-reactive protein; PCT, procalcitonin; PLT, platelet; HB, hemoglobin.

After the first session of TPE+CVVHDF treatment, the neutrophil-to-lymphocyte ratio (NLR), body temperature, and CRP showed different degrees of amelioration (**Figures 1A, D**). *Plasmodium* was found on a blood smear, with a density of 84.8 * 10^10^/L. The patient was diagnosed with severe falciparum malaria. On the third day, the second session of the TPE+CVVHDF treatment was conducted. Subsequently, only CVVHDF treatment was performed on the fourth and fifth days. Artemisinin (120 mg q12h on the first day, 120 mg qd for the following 9 days), along with intravenous injection of immunoglobulin (IVIG) and MP, was continued with the dosage adjusted according to the patient’s condition. The parameters indexed a severe condition, such as the A Physiology and Chronic Health Evaluation II (APACHE II) score and NLR, which gradually improved following the remission of parasitemia (**Figure 1A**). Almost all the laboratory parameters, such as CRP, PCT, and ALT, improved over the same period (**Figures 1B, D**). Hematological parameters such as HB and PLT gradually ameliorated (**Figure 1C**). On day 9, all indicators were close to normal. Finally, the patient was discharged on day 13 after admission.

### 3.2 Dynamic changes of *Plasmodium* counts

**Figure 2:**
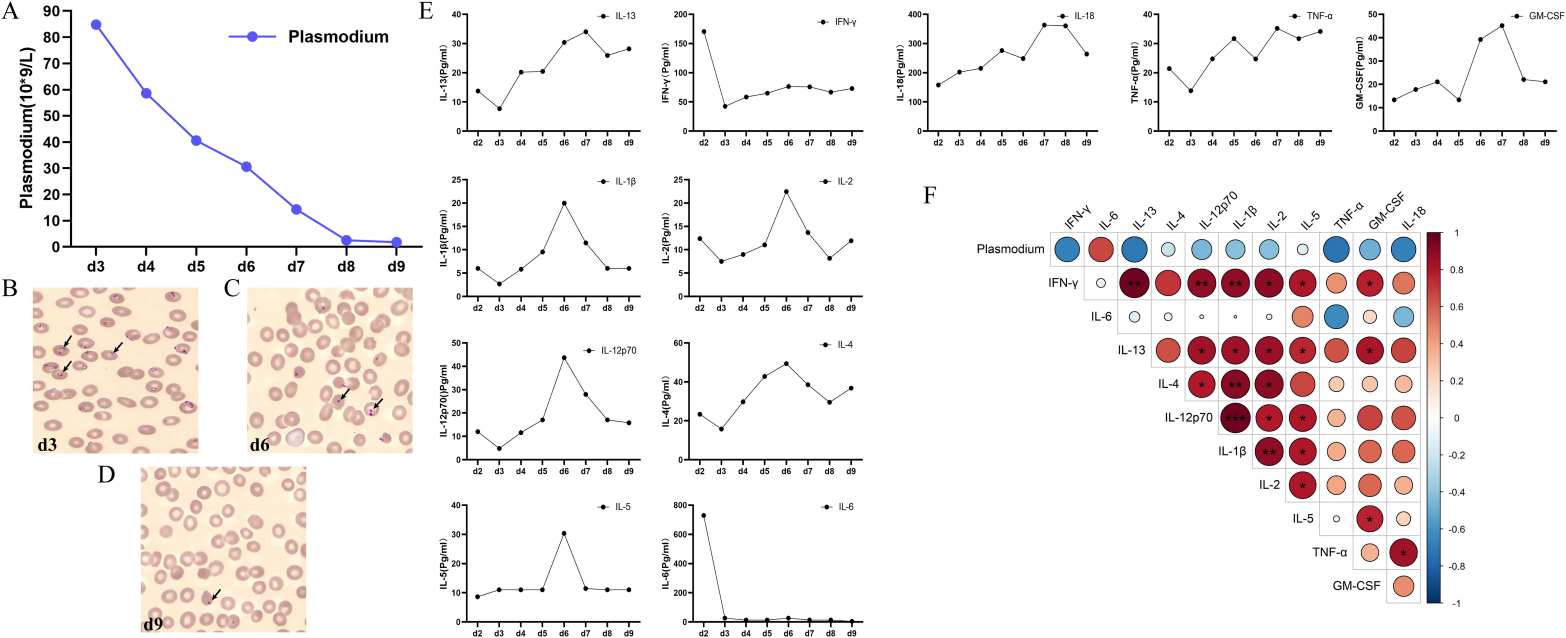
Dynamic changes and correlations between parasite count and cytokine concentrations. **(A)** The dynamic changes in parasite counts after admission. **(B–D)** Comparison of *Plasmodium* ring on d3, d6, and d9. **(E)** The dynamic changes of IFN-γ, IL-6, IL-13, IL-4, IL-12p 70, IL-1β, IL-2, IL-5, TNF-α, GM-CSF, and IL-13. **(F)** Correlation analysis of cytokines with *Plasmodium* from day 3 to day 9. *P < 0.05; **P < 0.01; **P < 0.01. IFN-γ, interferon-gamma; IL-6, interleukin-6; IL-13, interleukin-13; IL-4, interleukin-4; IL-12 p70, interleukin-12 p70; IL-13, interleukin-13; IL-1β, interleukin-1β; IL-2, interleukin-2; IL-5, interleukin-5; TNF-α, tumor necrosis factor-α; GM-CSF, granulocyte–macrophage colony-stimulating factor.

The total number of whole or residual *Plasmodium* ring bodies on the blood smear gradually decreased after confirmation of diagnosis (**Figures 2A–D**). On day 9, they were nearly cleared under microscopic examination (**Figure 2D**).

### 3.3 Dynamic changes of cytokine concentrations

Besides granulocyte–macrophage colony-stimulating factor (GM-CSF), several patterns of dynamic changes were found (**Figure 2E**). The first pattern dramatically elevated cytokines, with interferon-γ (IFN-γ) and interleukin-6 (IL-6) markedly increased at the onset of MODS. They decreased sharply after the first session of TPE+CVVHDF, from 70.38 to 42.47 pg/mL and 729.65 to 26.42 pg/mL, respectively. After that, the concentration of these two cytokines remained at a relatively low level. Interleukin-4 (IL-4), interleukin-13 (IL-13), interleukin-1β (IL-1β), and tumor necrosis factor-α (TNF-α) belonged to the second pattern, and their levels at the onset of MODS were lower than during recovery and on discharge. The third pattern of cytokines showed relatively lower concentrations at the onset of MODS and at discharge, while the top level of concentrations appeared mid-way through the recovery period. Interleukin-2 (IL-2), interleukin-5 (IL-5), and interleukin-12 p70 (IL-12 p70) belonged to the third pattern.

### 3.4 Correlation between *Plasmodium* counts and cytokine concentrations

The heatmap showed the Spearman correlation coefficient between the *Plasmodium* counts and cytokine concentrations (**Figure 2F**). After the first session of TPE+CVVHDF, all cytokines except IL-6 were found to exhibit negative trends with the amount of *Plasmodium*, although the results were not statistically significant. The majority of inflammatory and anti-inflammatory cytokines exhibited a positive correlation with each other.

### 3.5 Summary of *Plasmodium* infection cases

Although TPE as an adjunctive treatment for severe malaria lacks large-scale clinical trials, it has been mentioned in numerous case reports. We have compiled detailed information on the application of blood purification methods in malaria, including 29 case reports and 6 small cohort studies, and summarized them in **Table 1**. The results from all case reports showed that 25 patients infected with *P. falciparum* and 4 patients infected with *Plasmodium vivax* received blood purification-related treatments, with 19 of these patients undergoing TPE-based blood purification therapy. All patients fully recovered and were discharged. V. B. Chentsov and colleagues conducted a clinical cohort study involving 72 patients with severe malignant malaria infection, of which 48 received optimized ICU case management treatment. This included the early application of extracorporeal blood purification techniques (CVVHDF and TPE) and mechanical ventilation, aimed at preventing ischemia–reperfusion injury to the organs. The results showed that the cure rate in the trial group was 93.75%, the average ICU length of stay was reduced from 9.4 to 6.67 days, and the mortality rate decreased from 29.1% to 6.25% (Chentsov et al., 2020). The study by Bhadauria D. and R. Bambauer et al. also demonstrated the significant efficacy of TPE combined with CVVHDF in the treatment of severe malaria (Bambauer and Jutzler, 1985; Dharmendra et al., 2018). It is worth noting that TPE can still achieve remarkable effects when severe malaria is complicated by diseases or associated with complications such as thrombotic microangiopathy (TMA) (Dharmendra et al., 2018), Guillain–Barré syndrome (Sithinamsuwan et al., 2001), blackwater fever (Hira et al., 2023), leptospirosis (Sukhen et al., 2014), or purpura fulminans (Yasuyuki et al., 2007). Exchange transfusion is a specialized form of blood purification that includes processes such as red blood cell exchange and plasma exchange, targeting all blood components. We have summarized that 15 patients diagnosed with severe malaria were treated with exchange transfusion, and all patients eventually recovered, with 5 patients with cerebral malaria fully regaining consciousness within 3 days (Jingjing et al., 2024). In a case report, Ranjith K. N. et al. reviewed a case of a 24-year-old female patient with *P. vivax* infection complicated by TMA, noting that early identification of TMA and treatment with plasma exchange could facilitate earlier recovery (Ranjith et al., 2019). These results strongly suggest that timely TPE may have a positive effect on improving patient prognosis. It not only effectively removes bilirubin, liver enzymes, and creatinine from the plasma and improves systemic inflammatory response but also ameliorates adverse outcomes following malaria parasite clearance. Although the improvement of these laboratory parameters does not necessarily mean that TPE directly improves MODS, it holds great potential in reducing mortality associated with severe malaria.

**Table 1:** Summary of case reports on treating malaria with blood purification as an adjunctive therapy.

Reference	Number of cases	Sex	Age	*Plasmodium* species	Thrombocytopenia	Altered states of consciousness	Coagulation disorder	Acute kidney injury	Hyperbilirubinemia	Using artemisinin-based drugs	Mechanical ventilation	Blood purification methods	Survive
Fiona, M. et al., 2025 (Fiona et al., 2025)	1	Male	26	*Plasmodium falciparum*	Y	N	Y	Y	Y	Y	N	CRRT	Y
Rosanna Carmela, D.R. et al., 2025 (Rosanna Carmela et al., 2025)	1	Male	53	*Plasmodium falciparum*	NA	N	NA	N	Y	Y	N	EHA	Y
X, M. et al., 2025 (Ma et al., 2025)	1	Male	48	*Plasmodium falciparum*	Y	Y	N	Y	Y	Y	N	TPE+CVVHDF	Y
Daren Esteban Araque, G. et al., 2024 (Daren Esteban Araque et al., 2024)	1	Male	71	*Plasmodium vivax* malaria	Y	Y	Y	Y	Y	Y	N	TPE	Y
Pinar, G. et al., 2024 (Pinar et al., 2024)	1	Male	22	*Plasmodium falciparum*	Y	Y	NA	Y	Y	Y	N	Hemodialysis	Y
Rosana, G.S. et al., 2023 (Rosana et al., 2023)	1	Male	36	*Plasmodium falciparum*	Y	Y	NA	Y	Y	Y	N	TPE+CVVHDF+CPFA	Y
Hira, H. et al., 2023 (Hira et al., 2023)	1	Male	24	*Plasmodium falciparum*	Y	Y	Y	Y	Y	Y	N	TPE + hemodialysis	Y
Chentsov, V.B. et al., 2020 (Chentsov et al., 2020)	1	Male	49	*Plasmodium falciparum*	Y	Y	NA	Y	Y	Y	Y	TPE+CVVHDF	Y
Ratnadeep, G. et al., 2020 (Ratnadeep and Ajay, 2020)	1	Female	50	*Plasmodium falciparum*	Y	Y	N	Y	N	NA	N	TPE + hemodialysis	Y
Neil, R. et al., 2019 (Neil et al., 2019)	1	Female	53	*Plasmodium falciparum*	Y	Y	NA	Y	Y	N	N	Hemodialysis	Y
Ranjith K, N. et al., 2019 (Ranjith et al., 2019)	1	Female	24	*Plasmodium vivax malaria*	Y	N	NA	Y	Y	Y	N	Hemodialysis	Y
Cheryl L, M. et al., 2018 (Cheryl et al., 2018)	1	Male	18	*Plasmodium falciparum*	Y	Y	Y	Y	Y	Y	Y	RBC ET+TPE+CVVHDF	Y
Tsong-Yih, O. et al., 2017 (Tsong-Yih et al., 2017)	1	Female	23	*Plasmodium falciparum*	Y	Y	NA	Y	Y	Y	Y	TPE + hemodialysis	Y
Iee Ho, C. et al., 2016 (Iee Ho et al., 2016)	1	Male	15	*Plasmodium falciparum*	Y	N	NA	Y	Y	N	N	TPE + hemodialysis	Y
Najamus, S. et al., 2015 (Najamus et al., 2015)	1	Male	72	*Plasmodium falciparum*	N	N	NA	Y	Y	Y	N	RRT	Y
Keskar, V.S. et al., 2014 (Keskar et al., 2014)	1	Male	29	*Plasmodium vivax malaria*	Y	N	N	Y	Y	Y	N	TPE + hemodialysis	Y
Sukhen, S. et al., 2014 (Sukhen et al., 2014)	1	Female	24	*Plasmodium falciparum*	Y	Y	Y	Y	Y	Y	Y	TPE + hemodialysis	Y
Vivek Balkrishna, K. et al., 2014 (Vivek Balkrishna et al., 2014)	1	Male	12	*Plasmodium falciparum*	Y	Y	NA	Y	N	Y	N	Hemodialysis	Y
Biserka, T.-V. et al., 2013 (Biserka et al., 2013)	1	Male	56	*Plasmodium falciparum*	Y	N	NA	Y	Y	Y	N	Hemodialysis	Y
Hyun-Jung, L. et al., 2013 (Hyun-Jung et al., 2013)	1	Male	59	*Plasmodium vivax malaria*	Y	Y	Y	Y	N	N	Y	CVVHDF	Y
Kok Pin, Y. et al., 2012 (Kok Pin et al., 2012)	1	Male	49	*Plasmodium falciparum*	Y	N	NA	Y	Y	Y	N	Hemodiafiltration	Y
Yasuyuki, K. et al., 2007 (Yasuyuki et al., 2007)	1	Female	67	*Plasmodium falciparum*	Y	Y	Y	Y	Y	Y	Y	TPE + hemodialysis + hemofiltration	Y
Sithinamsuwan, P. et al., 2001 (Sithinamsuwan et al., 2001)	1	Female	28	*Plasmodium falciparum*	N	N	N	Y	N	N	Y	TPE	Y
L, M. et al., 1999 (Mathon et al., 1999)	1	Male	53	*Plasmodium falciparum*	Y	Y	Y	Y	Y	N	Y	ET (RBC+TPE) + hemodialysis	Y
P, J. et al., 1997 (Jacobs et al., 1997)	1	Male	25	*Plasmodium falciparum*	Y	N	Y	Y	NA	N	N	ET (whole blood + TPE)	Y
G, L. et al., 1992 (Lercari et al., 1992)	1	Male	53	*Plasmodium falciparum*	Y	Y	Y	Y	Y	N	N	ET (RBC+TPE) + hemodialysis	Y
U, S. et al., 1988 (Stuby et al., 1988)	1	Female	31	*Plasmodium falciparum*	Y	Y	Y	Y	Y	Y	Y	TPE + hemodialysis	Y
M U, H. et al., 1988 (Heim et al., 1988)	1	Male	42	*Plasmodium falciparum*	Y	Y	NA	Y	Y	N	N	TPE + ET + hemodialysis	Y
B, R. et al., 1988 (Rouvier et al., 1988)	1	Male	26	*Plasmodium falciparum*	Y	Y	Y	Y	Y	N	N	ET (RBC+TPE)	Y
Jingjing, Z. et al., 2024 (Jingjing et al., 2024)	8	8 Male	41.25 ± 7.94	*Plasmodium falciparum*	7 (87.5%)	5 (62.5%)	0 (0%)	3 (37.5%)	7 (87.5%)	NA	1 (1.25%)	ET (RBC+TPE) (8, 100%) RRT (1, 12.5%) CRRT (1, 12.5%) Hemodialysis (1, 12.5%)	8 (100%)
Chentsov, V.B. et al., 2020 (Chentsov et al., 2020)	48	38 Male/10 female	41.3± 3.97	*Plasmodium falciparum*	NA	20 (41.7%)	39 (81.2%)	N	4 (8.3%)	1 (2.1%)	20 (41.7%)	CVVHDF (37, 77.1%) TPE (14, 29.2%)	45 (93.75%)
Dharmendra, B. et al., 2018 (Dharmendra et al., 2018)	4	2 male/2 female	32.5 ± 6.18	*Plasmodium vivax malaria*	4 (100%)	0 (100%)	NA	4 (100%)	4 (100%)	4 (100%)	0 (100%)	TPE + hemodialysis (4, 100%)	4 (100%)
Spinello, A. et al., 2017 (Spinello et al., 2017)	12	7 male/5 female	42.17 ± 8.71	*Plasmodium falciparum*	12 (100%)	6 (50%)	NA	4 (33.3%)	10 (83.3%)	0 (0%)	5 (41.7%)	ET (RBC+TPE) (2, 16.7%); RRT (4, 33.3%)	12 (100%)
Rubina, N. et al., 2016 (Rubina, 2016)	109	66 male/43 female	33.49± 14.67	*Plasmodium vivax malaria*	NA	14 (12.84%)	15 (13.7%)	76 (69.72%)	42 (38.53%)	NA	8 (7.34%)	Hemodialysis (82, 75.22%)	69 (63.3%)
R, B. et al., 1985 (Bambauer and Jutzler, 1985)	3	2 male/1 female	40± 1.63	*Plasmodium falciparum*	3 (100%)	2 (66.7%)	2 (66.7%)	3 (100%)	3 (100%)	0 (0%)	NA	TPE + hemodialysis (2, 66.7%); hemodialysis (1, 33.3%)	2 (66.7%)

EHA, extracorporeal hemoadsorption; TPE, therapeutic plasma exchange; CVVHDF, continuous venovenous hemodiafiltration; CPFA, coupled plasma filtration adsorption; ET, exchange transfusion; RBC ET, red blood cell exchange transfusion; RRT, renal replacement therapy; CRRT, continuous renal replacement therapy; Y, yes; N, none; NA, information not applicable.

## 4 Discussion

Malaria is a protozoan disease transmitted through the bites of infected *Anopheles* mosquitoes, posing a significant public health burden. Current obstacles to national malaria elimination efforts are related to increasing drug resistance in the parasite, increasing insecticide resistance in its vectors, and human travel and migration. Of the five species of human malaria parasites, *P. falciparum* is responsible for the majority of severe malaria cases. The major complications of severe malaria include cerebral malaria, respiratory distress, acute kidney injury, severe anemia, or bleeding (Michael et al., 2014). With appropriate and timely treatment, the mortality rate of uncomplicated *P. falciparum* malaria (i.e., patients who can swallow medications and food) is <0.1%. However, once significant organ dysfunction develops or the total proportion of infected red blood cells increases to over 2%, the risk of mortality rises dramatically. Studies have shown that glycosylphosphatidylinositols from *Plasmodium* are an important pathogenic factor, capable of inducing cytokines such as TNF-α and interleukin-1 (IL-1) (Schofield and Hackett, 1993).

Much of the pathology in severe malaria is caused by excessive inflammatory responses, and it is currently believed that coagulation and fibrinolytic system disorders are mediated by elevated levels of certain cytokines. Pathogen infection causes immune cells to produce more cytokines, leading to a CS. CS is a life-threatening systemic inflammatory syndrome characterized by three main features: 1) increased cytokine levels in the bloodstream, 2) onset of systemic inflammation symptoms, and 3) occurrence of consequential dysfunction in the organs (Fajgenbaum and June, 2020). The occurrence of a CS, in turn, activates the coagulation system and promotes the development of acute respiratory distress syndrome (ARDS) and MODS. Autopsy studies in Malawi have revealed evidence of widespread systemic inflammation in multiple organs of deceased patients, suggesting that malaria deaths with coma may occur in the context of systemic inflammation (Rogerson et al., 1999). Therefore, evaluating the inflammation-mediated responses following severe malaria infection and maintaining the balance between pro-inflammatory and anti-inflammatory cytokines are crucial, and targeted therapies to address excessive pro-inflammatory responses in severe malaria may be an effective treatment strategy for improving prognosis.

In our study, the concentrations of two key cytokines, IL-6 and IFN-γ, dramatically rose to 729.65 and 170.38 pg/mL, respectively, at which time the APACHE II score reached peak level synchronously (**Figure 1A**). Also, the characteristics of common systemic inflammatory symptoms, such as fever, fatigue, and loss of appetite, were experienced by the patient. Secondary organ dysfunction, including ARDS and acute kidney injury (AKI), also appeared. In a word, CS was confirmed in the patient.

Pathologically aberrant elevated IL-6 and IFN-γ play an important role in the malignant cycle of producing excessive cytokines in CS (Clark et al., 2008; Fajgenbaum and June, 2020; Popa and Popa, 2021). It will lead to cytokine-driven MODS through prolonged activation of signaling pathways (Fajgenbaum and June, 2020). For IL-6, it is also the key factor correlated with acute inflammatory response, and our research also showed the coincidence of acute phase proteins such as CRP and PCT with IL-6. Previous studies also indicated that increasing concentrations of IL-6 were positively correlated with disease severity in patients infected with malaria (Mbengue et al., 2016; Wilairatana et al., 2022). Aberrantly accumulated IFN-γ also led to pathology and complications of severe malaria (Popa and Popa, 2021). These findings were consistent with our results. On the other hand, many pro-inflammatory cytokines, including IFN-γ, have been found to play a double role in malaria infection (Popa and Popa, 2021). After the first session of TPE+CVVDH, a negative correlation trend was found between IFN-γ, IL-2, IL-12 (p70), TNF-α, and parasite density (**Figure 2F**), which may be due to the complex biological mechanisms triggered by infection with *P. falciparum*. The immune response triggered by *Plasmodium* infection involves the interaction of various cytokines and immune cells, but this is consistent with the results that appropriately increasing pro-inflammatory cytokines will be beneficial for malaria control (Popa and Popa, 2021). The positive correlation between IFN-γ and anti-inflammatory or eosinophil-related cytokines such as IL-5 and IL-13 suggests that a balance of co-increasing pro-inflammatory and anti-inflammatory cytokines in a suitable range may benefit malaria clearance and prognosis.

TPE is a form of blood purification, and its mechanism of action involves mechanically removing pathogenic abnormal substances (such as autoantibodies, inflammatory mediators, and cytokines) from the plasma to reduce harmful substances and improve blood condition (Joan et al., 2020; Sowmya et al., 2020). The study found that TPE also has immunomodulatory effects, including the regulation of T cells, the stable shift from TH1/TH2 toward TH2, and the suppression of IL-2 and INF-γ production (Jeffrey, 2012). We searched for relevant studies on the use of blood purification methods for the treatment of malaria since 1985 and identified 29 case reports and 6 small cohort studies. Among the patients, 19 received TPE-based blood purification therapy. These patients were diagnosed with severe malaria in the early stage of infection, presenting with multi-organ dysfunction and severe inflammatory response, and all of them eventually made a full recovery and were discharged from the hospital. Therefore, we believe that TPE may be an effective adjuvant therapy for severe malaria, potentially improving patient survival and prognosis by mitigating the inflammatory response associated with severe malaria (Stuby et al., 1988; Keskar et al., 2014). It is worth noting that these studies did not explore the application of relevant therapeutic methods in severe malaria from the perspective of immune mechanisms. Therefore, we dynamically observed the changes in cytokine levels during the treatment process. On the basis of treatment effectiveness, we explored the immune mechanisms of the “TPE+CVVHDF” regimen in combating malaria complicated by cytokine storm through changes in cytokines. Previous experience in severe cases of H7N9 influenza infection and COVID-19 with acute MOF shows that TPE has a significant effect on improving metabolic disorders and organ dysfunction (Stegmayr et al., 2003; Liu et al., 2015; Zhang et al., 2021), because it can not only remove cytokines but also remove macromolecular substances in the circulation, such as bilirubin and creatinine in the plasma and other body metabolites (Hughes, 2002; Atan et al., 2013). This also provides evidence for the use of TPE in treating patients with severe and complex malaria (Sithinamsuwan et al., 2001; Ou et al., 2018). CVVHDF is a mixed purification mode that combines filtration and dialysis. It can reduce the CS through highly selective adsorption of small and medium molecular proteins. Although CVVHDF has a limited ability to remove cytokines, especially large molecules such as interleukin trimer, the combination with TPE can improve the management of metabolic disorders, fluid overload, and cardiovascular dysfunction (Hughes, 2002; Shiga et al., 2014).

Several studies have shown that the application of TPE or TPE in combination with other blood purification protocols, compared with traditional antimalarial therapy, can improve the survival rate and shorten the duration of hyperparasitemia and hospital stay in patients with severe and complicated falciparum malaria by targeting the CS (Chentsov et al., 2020). Exchange transfusion has also been mentioned in previous case reports, but it has a greater impact on the immune system, poses higher operational risks, and has a weaker ability to remove inflammatory mediators. Even though the treatment protocol of “TPE+CVVHDF” itself carries certain therapeutic risks, including the risk of infection, coagulation dysfunction, allergic reactions, electrolyte disturbances and acid–base imbalances, and even the possibility of exacerbating cytokine storm and worsening organ damage, there have been almost no adverse events that have been definitively attributed to the application of this treatment protocol in the treatment of severe falciparum malaria according to relevant research reports over the past few decades. In this study, we closely monitored the patient’s clinical indicators and vital signs, including routine blood tests, biochemical functions, inflammatory markers, and coagulation functions, until the condition significantly improved, and no obvious adverse reactions or complications occurred during the entire treatment process. However, we still recommend that the use of this protocol to treat patients with falciparum malaria complicated by cytokine storm should be considered with caution. Studies have shown that glucocorticoids have an effective role in combating CS (Frey, 2017; Organization WH, 2022), and when TPE is used in combination with glucocorticoids, it can significantly shorten the remission time of CS from 12 to 6 days (Kamran et al., 2021). It is also confirmed in our study, after the first session of TPE+CVVHDF, that the cytokines sharply remitted to a relatively low level, and CS was resolved immediately. However, this study also has limitations. As the area is not an endemic region for malaria, and *P. falciparum* infection is a rare case, the study only involved the treatment course of one patient. Despite the samples being collected in a row and the cytokine data being analyzed from different aspects, the potential impact of mixed factors such as the application of glucocorticoids and immunoglobulin on immune regulation still existed. Therefore, larger-scale randomized clinical trials are still needed in the future to systematically verify the immune mechanism of this regimen in the treatment of severe falciparum malaria patients so as to provide more reliable evidence for clinical treatment.

## 5 Conclusion

**Figure 3:**
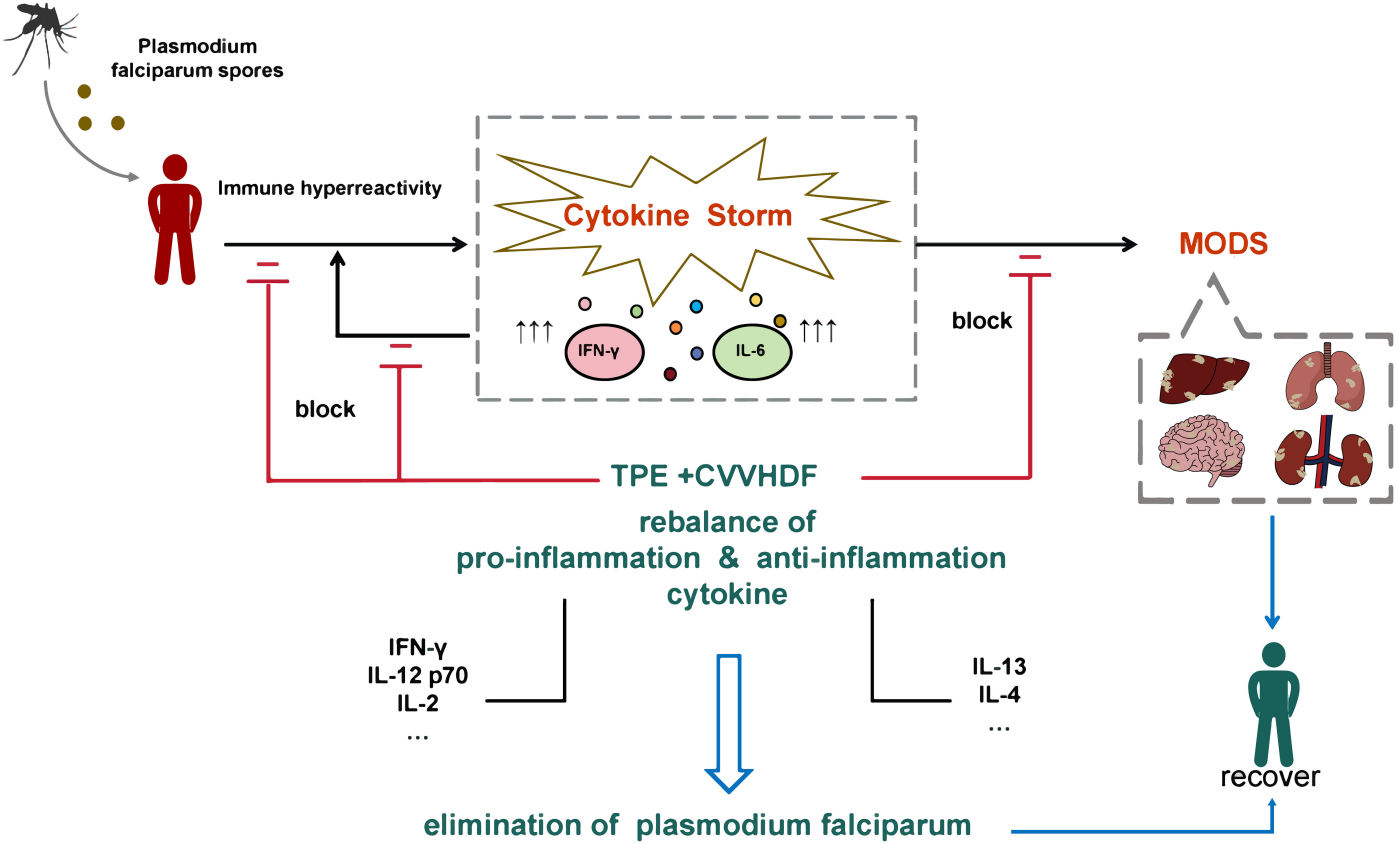
Diagram of the TPE+CVVHDF-based therapy strategy successfully rescuing the severe falciparum malaria patient experiencing cytokine storm-driven MODS. TPE, therapeutic plasma exchange; CVVHDF, continuous venovenous hemodiafiltration; MODS, multiple organ dysfunction syndrome.

The advent of artemisinin-based drugs has significantly reduced mortality rates in severe malaria. However, selecting effective antiparasitic drugs is no longer the sole concern in treating severe malaria. Due to the increasing number of imported severe tropical malaria cases in recent years and the variability of initial symptoms, accurate diagnosis is particularly important. Early diagnosis and timely, effective treatment of falciparum malaria can prevent severe sequelae and death and reduce the risk of further parasite transmission. For patients in a coma, experiencing CS, or showing signs of MOF, it is recommended to initiate TPE combined with CVVHDF treatment as early as possible. However, this should be done with caution, taking into account the patient’s underlying health conditions and comorbidities to prevent serious treatment risks and complications. Our research suggests that the strategy of TPE combined with CVVHDF therapy shows promise in targeting CS and rescuing severe falciparum malaria patients with MODS (**Figure 3**).

## Data Availability

The original contributions presented in the study are included in the article/**Supplementary Material**. Further inquiries can be directed to the corresponding authors.
