# Correction: The blood purification therapy-based strategy effectively rescued the severe falciparum malaria patient experiencing cytokine storm-driven MODS

**DOI:** 10.3389/fcimb.2026.1795192

**Published:** 2026-02-20

**Authors:** Ying Sun, Xi Huang, Bao Hong, Jinyuan Song, Ying Lu, Jindi Ma, Xiaojing Wang, Wenqing Hu, Yimin Zhang, Hua Xuan

**Affiliations:** 1 State Key Laboratory for Diagnosis and Treatment of Infectious Diseases, National Clinical Research Center for Infectious Diseases, National Medical Center for Infectious Diseases, The First Affiliated Hospital, Zhejiang University School of Medicine, Hangzhou, China; 2 Department of Electrocardiogram, The First Affiliated Hospital, School of Medicine, Zhejiang University, Hangzhou, China; 3 Department of Infectious Diseases, The Affiliated Haining Hospital, Jiaxing University, Jiaxing, China; 4 Department of Clinical Laboratory, The Affiliated Haining Hospital, Jiaxing University, Jiaxing, China; 5 Emergency Department, The Affiliated Haining Hospital, Jiaxing University, Jiaxing, China; 6 Department of Infectious Diseases, Affiliated Hospital of Jiaxing University, The First Hospital of Jiaxing, Jiaxing, China

**Keywords:** continuous veno-venous hemodiafiltration, cytokine storm, falciparum malaria, multiple organ dysfunction syndrome, therapeutic plasma exchange

Affiliation “Department of Infectious Diseases, Affiliated Hospital of Jiaxing University, The First Hospital of Jiaxing, Jiaxing, China” was erroneously given as “Department of Infectious Diseases, Jiaxing no.1 People’s Hospital, Jiaxing, China”.

The original version of this article has been updated.

